# Supplementary material for: CoRNeA: A Pipeline to Decrypt the Inter-Protein Interfaces from Amino Acid Sequence Information
Source: Biomolecules. 2020 Jun 22;10(6):938. doi: 10.3390/biom10060938 (PMC7356028; doi:10.3390/biom10060938)
Supplement: Supplementary file 1 [file biomolecules-10-00938-s001.pdf]

**Supplementary Material**

# CoRNeA: A pipeline to decrypt the inter protein interfaces from amino acid sequence information

Kriti Chopra[1], Bhawna Burdak[1], Kaushal Sharma[2], Ajit Kembhavi[2], Shekhar C. Mande[3] and Radha Chauhan[1*]

1- National Centre for Cell Science, Pune.

2- Inter University Centre for Astronomy and Astrophysics, Pune

3- Council of Scientific and Industrial Research (CSIR), New Delhi

*__Corresponding Author__:

Dr. Radha Chauhan, Scientist 'E', National Centre for Cell Science, S.P. Pune University Campus, Ganeshkhind, Pune 411007, Maharashtra, India.

Email: radha.chauhan@nccs.res.in

Phone: +91-20-25708255

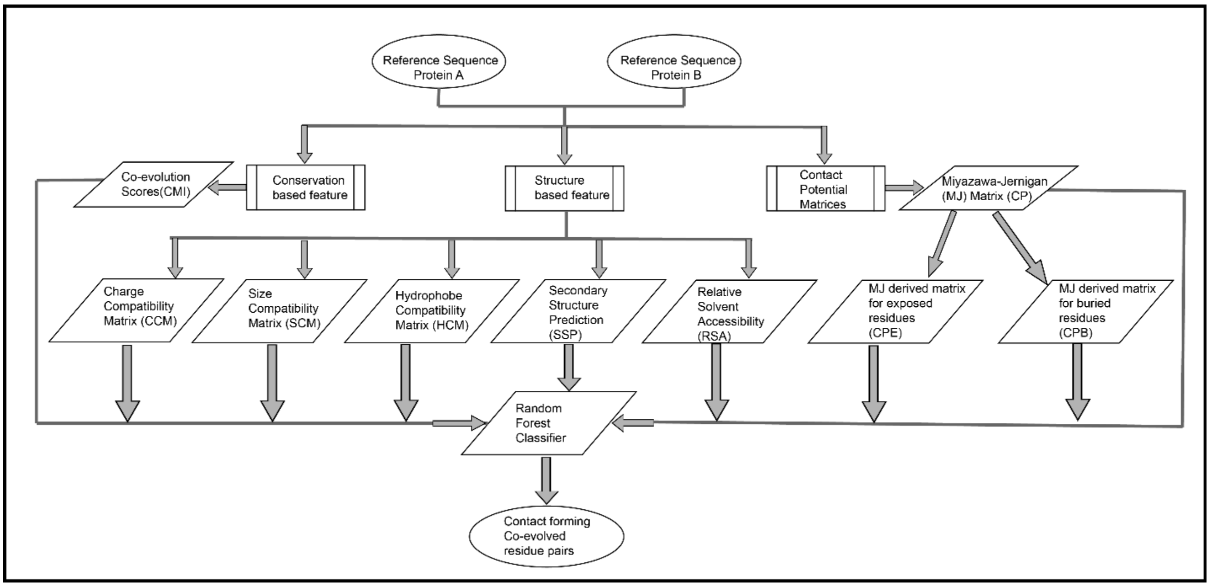


**Figure S1: Flowchart depicting the feature generation for predicting pair of protein-protein interaction interface residues**

**Table S1: Numeric Coding for amino acids used for co-evolution score calculations**

| **Amino Acid** | **Numeric Coding** |
|---|---|
| V (Valine) | 1 |
| I (Isoleucine) | 2 |
| L (Leucine) | 3 |
| M (Methionine) | 4 |
| F (Phenylalanine) | 5 |
| W (Tryptophan) | 6 |
| Y (Tyrosine) | 7 |
| S (Serine) | 8 |
| T (Threonine) | 9 |
| N (Asparagine) | 10 |
| Q (Glutamine) | 11 |
| H (Histidine) | 12 |
| K (Lysine) | 13 |
| R (Arginine) | 14 |
| D (Aspartic Acid) | 15 |
| E (Glutamic acid) | 16 |
| A (Alanine) | 17 |
| G (Glycine) | 18 |
| P (Proline) | 19 |
| C (Cysteine) | 20 |
| - (Gap) | 21 |
| X (Non-Standard Amino Acid) | 22 |

**Table S2: Comparison of known methods for PPI interface prediction with the new hybrid method**

| **Interface residues (PISA)** | | | **Various algorithms for finding contacts** | | | | |
|---|---|---|---|---|---|---|---|
| **Nup107** | **Nup133** | **Distance(Å)** | **MI** | **DCA** | **Evfold** | **SCA** | **New Method (CMI)** |
| **D 879** | **T 696** | 3.37 | 0.211 | 0.0139 | 0.0335 | 0.1601 | **0.804** |
| **S 822** | **K 975** | 2.78 | 0.117 | 0.00569 | 0.0148 | 0.041 | **0.591** |
| **E 884** | **K 975** | 2.69 | 0.117 | 0.00063 | 0.0135 | 0.0878 | **0.524** |
| **D 917** | **K 966** | 2.53 | 0.0051 | 0.0031 | 0.0083 | 0.0497 | **0.642** |
| **Y 921** | **K 966** | 3.37 | 0.1108 | 0.005 | 0.0193 | 0.1595 | **0.364** |
| **E 922** | **R 962** | 3.18 | 0.389 | 0.0094 | 0.0129 | 0.1922 | **0.342** |
| **K 894** | **D 982** | 3.82 | 0.174 | 0.0316 | 0.0032 | 0.0577 | **0.371** |
| **R 898** | **A 980** | 3.28 | 0.0881 | 0.0063 | 0.0161 | 0.0101 | **0.233** |
| **Q 902** | **Q 944** | 3.35 | 0.4174 | 0.0126 | 0.0064 | 0.378 | **0.159** |

The interface residues for a test case as predicted by PISA. All values are rescaled between 0 and 1. MI: Mutual information, DCA: Direct Coupling Analysis, SCA: Statistical Coupling Analysis.

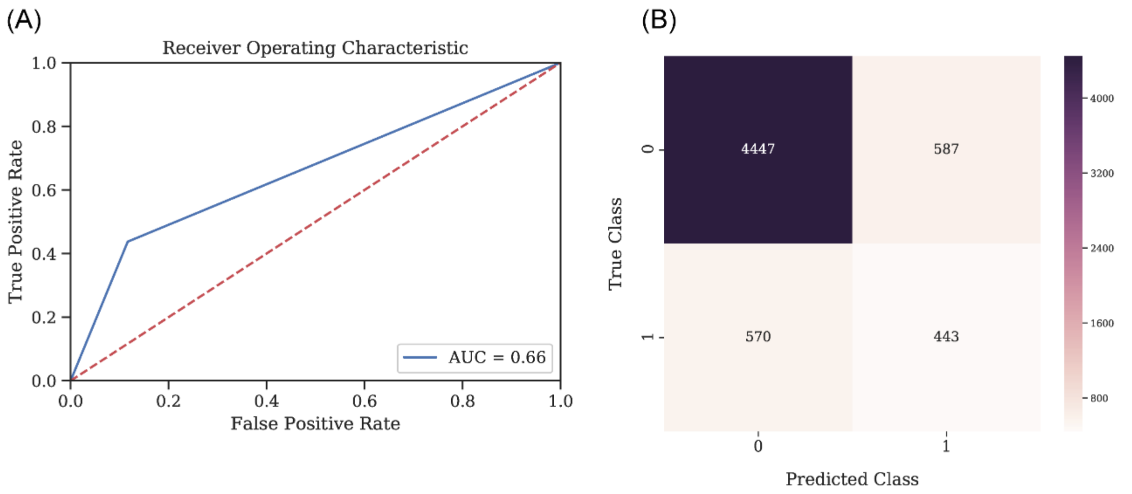


**Figure S2: Statistics for the Random Forest Classifier Model for predicting contact forming residue pairs without environmental features. (**A) Receiver-operator curve (ROC) depicting Area under the curve (AUC) as 0.66 when the model is tested on the 75:25 data split. (B) Confusion matrix for the tested model on 75:25 data split with a final accuracy of 80%

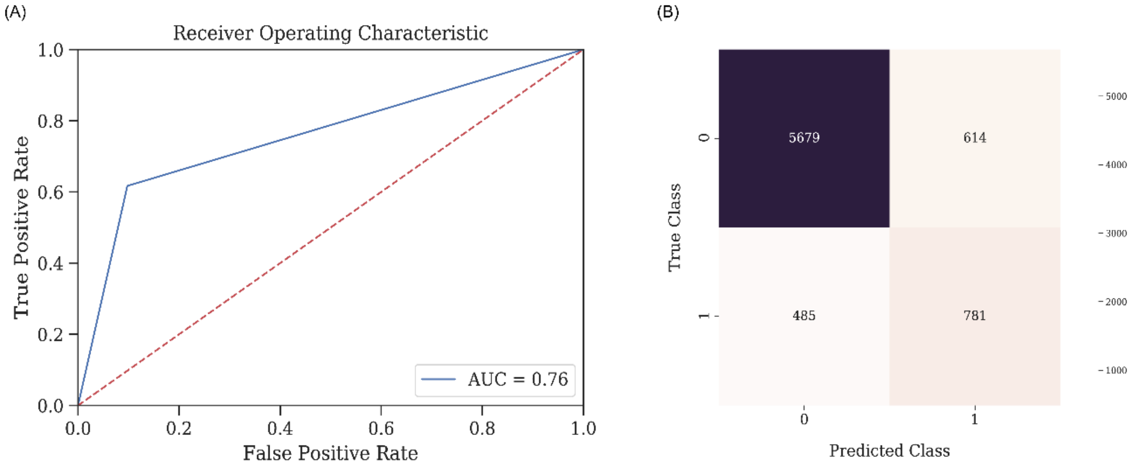


**Figure S3: Statistics for the Random Forest Classifier Model for predicting contact forming residue pairs with environmental features. (**A) Receiver-operator curve (ROC) depicting Area under the curve (AUC) as 0.76 when the model is tested on the 75:25 data split. (B) Confusion matrix for the tested model on 75:25 data split with a final accuracy of 86%

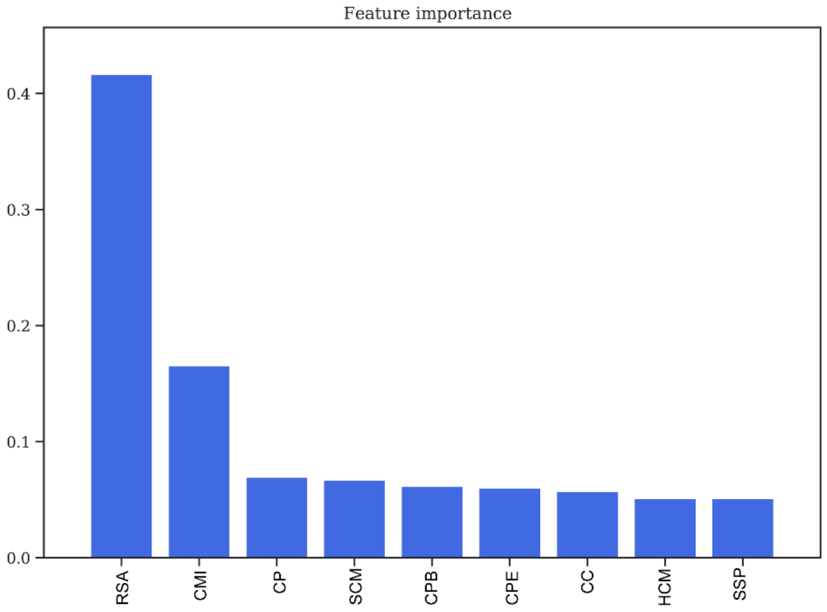


**Figure S4: Feature Importance obtained from Random Forest Classifier without environmental features.**

Relative Solvent Accessibility (RSA) and Co-evolution Scores (CMI) as two of the most important features in training the model. **RSA:** Relative Solvent Accessibility. **CMI:** Conditional Mutual Information. **CP:** Contact Potential. **SCM:** Structure Compatibility Matrix. **CPB:** Contact Potential for Buried residues. **CPE:** Contact Potential for Exposed residues. **CC:** Charge Compatibility. **HCM:** Hydropathy Compatibility Matrix. **SSP:** Secondary Structure Prediction.

**Table S3: Evaluation of different kernel matrix derived random forest classifier on different test datasets**

| PDB ID | Type of secondary structure | Best Kernel Matrix | Number of true positive labelled | Actual true positives predicted with best kernel matrix |
|---|---|---|---|---|
| 1GCQ | Loop:Loop<br>Loop:Sheet | 5*5 | 81 | 25 |
| 1Y8R | Helix:Helix<br>Loop:Loop | 3*3 | 157 | 23 |
| 4YDU | Helix:Helix | 3*3 | 86 | 23 |
| 5YVT | Helix:Helix<br>Sheet:Sheet<br>Loop:Loop | 5*5 | 164 | 64 |
| 3CQC | Helix:Helix | 3*3 | 48 | 13 |

**Table S4: Leave-one-out method for testing random forest component of CoRNeA**

| Protein Complex | Accuracy (%) | Precision (macro) | Recall (macro) | F1 score (macro) | Precision (weighted) | Recall (weighted) | F1 score (weighted) | AUC$_{ROC}$ |
|---|---|---|---|---|---|---|---|---|
| 1A2K | 82 | 0.51 | 0.75 | 0.47 | 0.99 | 0.82 | 0.89 | 0.75 |
| 1B6C | 95 | 0.50 | 0.48 | 0.49 | 1.00 | 0.95 | 0.97 | 0.484 |
| 1BUH | 84 | 0.50 | 0.51 | 0.46 | 0.99 | 0.84 | 0.91 | 0.51 |
| 1E6E | 98 | 0.50 | 0.53 | 0.50 | 0.99 | 0.98 | 0.98 | 0.53 |
| 1E 96 | 84 | 0.51 | 0.76 | 0.47 | 1.00 | 0.84 | 0.91 | 0.76 |
| 1GRN | 91 | 0.51 | 0.69 | 0.49 | 1.00 | 0.91 | 0.95 | 0.69 |
| 1GXD | 90 | 0.50 | 0.60 | 0.48 | 1.00 | 0.90 | 0.95 | 0.6 |
| 1H1V | 87 | 0.50 | 0.63 | 0.47 | 1.00 | 0.87 | 0.93 | 0.63 |
| 1H9D | 78 | 0.50 | 0.42 | 0.44 | 0.98 | 0.78 | 0.87 | 0.42 |
| 1HE8 | 93 | 0.50 | 0.66 | 0.48 | 1.00 | 0.93 | 0.96 | 0.66 |
| 1I2M | 91 | 0.50 | 0.58 | 0.48 | 0.99 | 0.91 | 0.95 | 0.58 |
| 1I4D | 84 | 0.50 | 0.60 | 0.46 | 1.00 | 0.84 | 0.91 | 0.6 |
| 1IB1 | 91 | 0.50 | 0.57 | 0.48 | 0.99 | 0.91 | 0.95 | 0.57 |
| 1IBR | 91 | 0.50 | 0.74 | 0.48 | 1.00 | 0.91 | 0.95 | 0.74 |
| 1JWH | 95 | 0.50 | 0.47 | 0.49 | 1.00 | 0.95 | 0.97 | 0.47 |
| 1K5D | 93 | 0.50 | 0.53 | 0.49 | 0.98 | 0.93 | 0.95 | 0.53 |
| 1KTZ | 86 | 0.50 | 0.43 | 0.46 | 0.99 | 0.86 | 0.92 | 0.43 |
| 1LFD | 89 | 0.52 | 0.75 | 0.50 | 0.99 | 0.89 | 0.93 | 0.75 |
| 1NVU | 96 | 0.50 | 0.54 | 0.50 | 0.99 | 0.96 | 0.98 | 0.54 |
| 1NW9 | 98 | 0.50 | 0.50 | 0.50 | 0.99 | 0.98 | 0.98 | 0.5 |
| 1OPH | 97 | 0.50 | 0.55 | 0.49 | 1.00 | 0.97 | 0.98 | 0.55 |
| 1RV6 | 78 | 0.50 | 0.55 | 0.44 | 0.99 | 0.78 | 0.87 | 0.55 |
| 1S1Q | 69 | 0.51 | 0.74 | 0.43 | 0.99 | 0.69 | 0.80 | 0.74 |
| 1WQ1 | 90 | 0.51 | 0.82 | 0.49 | 1.00 | 0.90 | 0.95 | 0.82 |
| 1XD3 | 80 | 0.50 | 0.62 | 0.46 | 0.99 | 0.80 | 0.88 | 0.62 |
| 1XQS | 94 | 0.51 | 0.59 | 0.50 | 0.99 | 0.94 | 0.96 | 0.59 |
| 1Z0K | 87 | 0.51 | 0.63 | 0.49 | 0.98 | 0.87 | 0.92 | 0.63 |
| 2C0L | 96 | 0.50 | 0.54 | 0.5 | 0.99 | 0.96 | 0.98 | 0.54 |
| 2HLE | 95 | 0.51 | 0.56 | 0.51 | 0.99 | 0.95 | 0.97 | 0.56 |
| 2OOB | 70 | 0.50 | 0.49 | 0.42 | 0.98 | 0.70 | 0.82 | 0.49 |
| 2OZA | 96 | 0.50 | 0.49 | 0.49 | 1.00 | 0.96 | 0.98 | 0.49 |
| 2PCC | 84 | 0.50 | 0.74 | 0.46 | 1.00 | 0.84 | 0.91 | 0.74 |
| 3AAA | 92 | 0.50 | 0.55 | 0.49 | 0.99 | 0.92 | 0.95 | 0.55 |
| 3CPH | 90 | 0.50 | 0.71 | 0.48 | 1.00 | 0.90 | 0.95 | 0.71 |
| 3DAW | 87 | 0.50 | 0.65 | 0.47 | 1.00 | 0.87 | 0.93 | 0.65 |
| 3F1P | 94 | 0.50 | 0.47 | 0.48 | 0.99 | 0.94 | 0.96 | 0.47 |
| 3H2V | 90 | 0.50 | 0.53 | 0.48 | 0.99 | 0.90 | 0.94 | 0.53 |
| 3K75 | 96 | 0.50 | 0.50 | 0.49 | 1.00 | 0.96 | 0.98 | 0.5 |
| 3R9A | 98 | 0.50 | 0.58 | 0.50 | 1.00 | 0.98 | 0.99 | 0.58 |
| 5C3L_AB | 94 | 0.50 | 0.57 | 0.49 | 1.00 | 0.94 | 0.97 | 0.57 |
| 5C3L_AC | 88 | 0.50 | 0.52 | 0.49 | 0.97 | 0.88 | 0.92 | 0.52 |
| 5C3L_BC | 91 | 0.50 | 0.55 | 0.48 | 0.99 | 0.91 | 0.95 | 0.55 |

**Table S5: Testing of 42 protein complexes on 42 complex trained model**

| Protein Complex | Accuracy (%) | Precision (macro) | Recall (macro) | F1 score (macro) | Precision (weighted) | Recall (weighted) | F1 score (weighted) | AUC $_{ROC}$ |
|---|---|---|---|---|---|---|---|---|
| 1A2K | 82 | 0.51 | 0.89 | 0.48 | 1.00 | 0.97 | 0.89 | 0.89 |
| 1B6C | 96 | 0.52 | 0.94 | 0.52 | 1.00 | 0.96 | 0.98 | 0.94 |
| 1BUH | 84 | 0.52 | 0.87 | 0.49 | 0.99 | 0.84 | 0.91 | 0.87 |
| 1E6E | 98 | 0.55 | 0.88 | 0.59 | 1.00 | 0.98 | 0.99 | 0.88 |
| 1E 96 | 86 | 0.51 | 0.91 | 0.48 | 1.00 | 0.86 | 0.92 | 0.91 |
| 1GRN | 92 | 0.52 | 0.9 | 0.51 | 1.00 | 0.92 | 0.96 | 0.9 |
| 1GXD | 93 | 0.51 | 0.89 | 0.49 | 1.00 | 0.93 | 0.96 | 0.89 |
| 1H1V | 91 | 0.5 | 0.86 | 0.48 | 1.00 | 0.91 | 0.95 | 0.86 |
| 1H9D | 79 | 0.52 | 0.83 | 0.48 | 0.99 | 0.79 | 0.88 | 0.83 |
| 1HE8 | 94 | 0.51 | 0.92 | 0.49 | 1.00 | 0.94 | 0.97 | 0.92 |
| 1I2M | 89 | 0.51 | 0.91 | 0.5 | 1.00 | 0.89 | 0.94 | 0.91 |
| 1I4D | 85 | 0.51 | 0.88 | 0.47 | 1.00 | 0.85 | 0.91 | 0.88 |
| 1IB1 | 87 | 0.51 | 0.89 | 0.49 | 1.00 | 0.87 | 0.93 | 0.89 |
| 1IBR | 94 | 0.51 | 0.92 | 0.5 | 1.00 | 0.94 | 0.97 | 0.92 |
| 1JWH | 96 | 0.51 | 0.9 | 0.51 | 1.00 | 0.96 | 0.98 | 0.9 |
| 1K5D | 85 | 0.52 | 0.89 | 0.5 | 0.99 | 0.85 | 0.91 | 0.89 |
| 1KTZ | 84 | 0.52 | 0.89 | 0.49 | 0.99 | 0.84 | 0.91 | 0.89 |
| 1LFD | 88 | 0.53 | 0.92 | 0.52 | 0.99 | 0.88 | 0.93 | 0.92 |
| 1NVU | 95 | 0.52 | 0.92 | 0.53 | 1.00 | 0.95 | 0.97 | 0.92 |
| 1NW9 | 93 | 0.54 | 0.92 | 0.55 | 0.99 | 0.93 | 0.96 | 0.92 |
| 1OPH | 98 | 0.52 | 0.94 | 0.54 | 1.00 | 0.98 | 0.99 | 0.94 |
| 1RV6 | 79 | 0.51 | 0.86 | 0.46 | 0.99 | 0.79 | 0.88 | 0.86 |
| 1S1Q | 67 | 0.52 | 0.84 | 0.43 | 0.99 | 0.67 | 0.79 | 0.84 |
| 1WQ1 | 91 | 0.51 | 0.94 | 0.49 | 1.00 | 0.91 | 0.95 | 0.94 |
| 1XD3 | 81 | 0.51 | 0.87 | 0.47 | 0.99 | 0.81 | 0.89 | 0.87 |
| 1XQS | 93 | 0.52 | 0.89 | 0.53 | 1.00 | 0.93 | 0.96 | 0.89 |
| 1Z0K | 87 | 0.53 | 0.89 | 0.53 | 0.99 | 0.87 | 0.92 | 0.89 |
| 2C0L | 96 | 0.53 | 0.89 | 0.54 | 1.00 | 0.96 | 0.98 | 0.89 |
| 2HLE | 94 | 0.54 | 0.91 | 0.56 | 0.99 | 0.94 | 0.96 | 0.91 |
| 2OOB | 70 | 0.51 | 0.81 | 0.44 | 0.99 | 0.70 | 0.81 | 0.81 |
| 2OZA | 96 | 0.52 | 0.92 | 0.53 | 1.00 | 0.96 | 0.98 | 0.92 |
| 2PCC | 90 | 0.51 | 0.92 | 0.49 | 1.00 | 0.90 | 0.95 | 0.92 |
| 3AAA | 88 | 0.52 | 0.88 | 0.51 | 0.99 | 0.88 | 0.93 | 0.88 |
| 3CPH | 92 | 0.51 | 0.94 | 0.49 | 1.00 | 0.92 | 0.95 | 0.94 |
| 3DAW | 89 | 0.51 | 0.91 | 0.49 | 1.00 | 0.89 | 0.94 | 0.91 |
| 3F1P | 95 | 0.53 | 0.87 | 0.54 | 1.00 | 0.95 | 0.97 | 0.87 |
| 3H2V | 89 | 0.52 | 0.89 | 0.52 | 0.99 | 0.89 | 0.94 | 0.89 |
| 3K75 | 97 | 0.52 | 0.92 | 0.53 | 1.00 | 0.97 | 0.98 | 0.92 |
| 3R9A | 98 | 0.52 | 0.92 | 0.53 | 1.00 | 0.98 | 0.99 | 0.92 |
| 5C3L_AB | 94 | 0.51 | 0.92 | 0.5 | 1.00 | 0.94 | 0.97 | 0.92 |
| 5C3L_AC | 68 | 0.52 | 0.8 | 0.44 | 0.99 | 0.68 | 0.80 | 0.8 |
| 5C3L_BC | 82 | 0.51 | 0.85 | 0.47 | 1.00 | 0.82 | 0.90 | 0.85 |

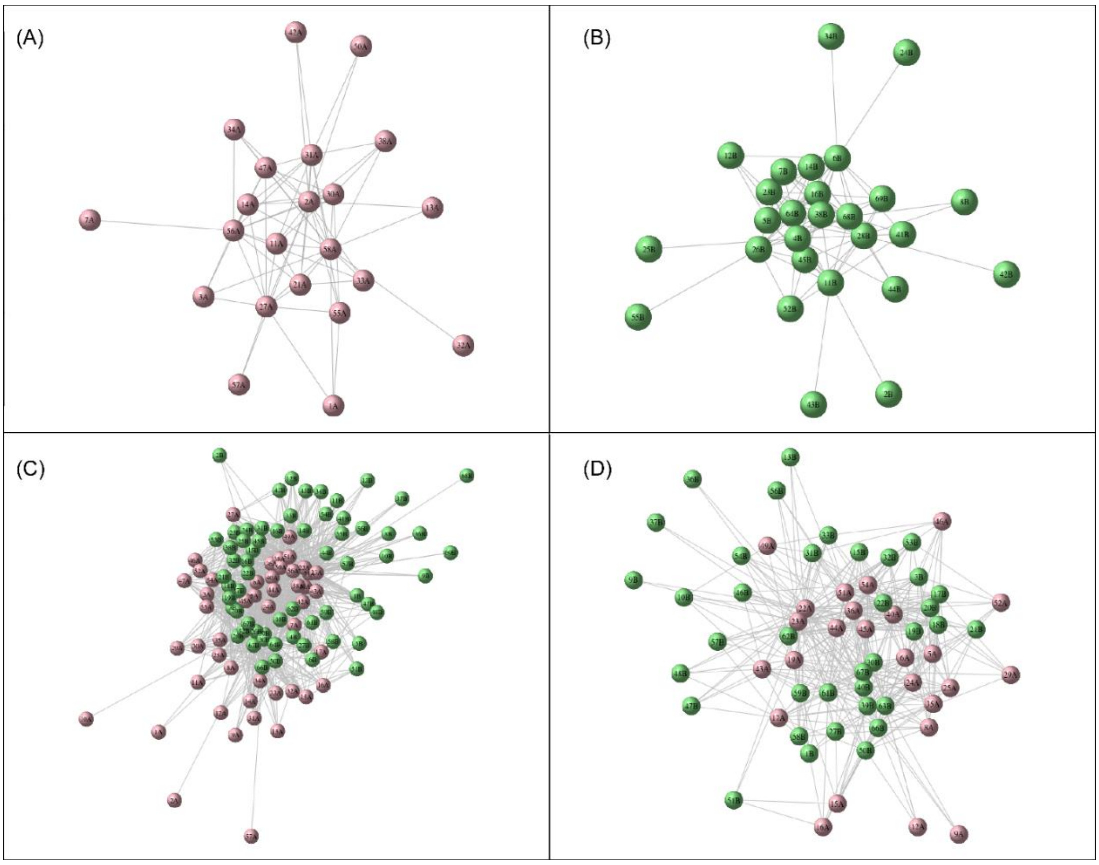


**Figure S5: Network analysis for PDB ID 1GCQ.** (A) Intra-protein network for Chain A/B of 1GCQ obtained from top 5% co-evolving intra residue pairs. (B) Intra-protein network for Chain C of 1GCQ obtained from top 5% co-evolving intra residue pairs. (C) Inter-protein network for 1GCQ obtained from random forest classifier. (D) Inter-protein network for 1GCQ after removing intra-protein network nodes and all nodes having relative solvent accessibility as 0.

**Table S6: Pairwise true contacts predicted for PDB ID 1GCQ Chain A with Chain C and Chain B with Chain C within a distance cutoff of 10 Å.**

| Residue number (Chain A) | Residue number (Chain C) | Convolution Value | Distance (Å) | Residue number (Chain B) | Residue number (Chain C) | Convolution Value | Distance (Å) |
|---|---|---|---|---|---|---|---|
| 208 | 612 | 7 | 3.53 | 179 | 652 | 7 | 3.3 |
| 192 | 611 | 7 | 3.6 | 165 | 655 | 8 | 4.66 |
| 208 | 611 | 8 | 3.62 | 179 | 655 | 9 | 6.7 |
| 194 | 608 | 7 | 3.7 | 164 | 657 | 7 | 7.2 |
| 209 | 607 | 8 | 3.7 | 179 | 653 | 7 | 7.5 |
| 209 | 610 | 11 | 3.9 | 179 | 654 | 8 | 8.9 |
| 193 | 610 | 9 | 4 | 179 | 629 | 8 | 9.8 |
| 193 | 611 | 7 | 4.17 | | | | |
| 208 | 610 | 9 | 4.39 | | | | |
| 209 | 609 | 11 | 4.78 | | | | |
| 165 | 608 | 7 | 4.8 | | | | |
| 209 | 611 | 9 | 4.9 | | | | |
| 209 | 608 | 9 | 5.13 | | | | |
| 207 | 611 | 8 | 5.2 | | | | |
| 209 | 651 | 7 | 6.8 | | | | |
| 164 | 607 | 9 | 7.15 | | | | |
| 193 | 609 | 9 | 7.3 | | | | |
| 207 | 610 | 9 | 7.47 | | | | |
| 164 | 608 | 11 | 7.49 | | | | |
| 179 | 606 | 9 | 7.6 | | | | |
| 192 | 609 | 9 | 7.7 | | | | |
| 209 | 612 | 7 | 7.8 | | | | |
| 179 | 607 | 12 | 8.5 | | | | |
| 165 | 609 | 8 | 8.7 | | | | |
| 193 | 608 | 7 | 8.8 | | | | |
| 165 | 610 | 7 | 8.9 | | | | |
| 209 | 653 | 7 | 9.3 | | | | |
| 192 | 608 | 7 | 9.6 | | | | |
| 179 | 608 | 12 | 9.8 | | | | |

## Table S7: Top 10% pairs predicted for Nup93-Nup205

| Nup205 | Nup93 | Convolution Score | No of pairs in the predicted regions |
|---|---|---|---|
| 1932-1936 | 86-99 | 272 | 57 |
| 1932-1936 | 101-117 | 234 | 54 |
| 1013-1014 | 86-109 | 100 | 30 |
| 1945-1948 | 44-48 | 82 | 16 |
| 1801-1805 | 44-48 | 71 | 15 |
| 749-751 | 86-97 | 66 | 18 |
| 1935-1939 | 448-452 | 65 | 16 |
| 1928-1930 | 87-94 | 65 | 17 |
| 682-684 | 109-115 | 63 | 21 |
| 1937-1940 | 44-48 | 63 | 14 |
| 1696-1700 | 44-48 | 59 | 15 |
| 1250-1252 | 87-93 | 55 | 17 |
| 1250-1252 | 109-113 | 45 | 15 |
